# Prevalence and social environment of cigarette smoking in Cyprus youth

**DOI:** 10.1186/1471-2458-8-190

**Published:** 2008-06-02

**Authors:** Costas A Christophi, Ourania Kolokotroni, Hillel R Alpert, Charles W Warren, Nathan R Jones, Philip Demokritou, Gregory N Connolly

**Affiliations:** 1 Cyprus International Institute for the Environment and Public Health in association with Harvard School of Public Health, Nicosia, Cyprus; 2 Department of Environmental Health, Harvard School of Public Health, Boston, MA, USA; 3 The Biostatistics Center, The George Washington University, Rockville, MD, USA; 4 Division of Public Health Practice, Harvard School of Public Health, Boston, MA, USA; 5 Office for Smoking and Health, Centers for Disease Control and Prevention, Atlanta, GA, USA

## Abstract

**Background:**

Tobacco use is the single most preventable cause of morbidity and mortality in humans. Limited data exist regarding the extent of the problem among Cyprus youth. We use the Global Youth Tobacco Survey to assess the prevalence of cigarette smoking among middle and high school students as well as the social environment in which this is taking place.

**Methods:**

The survey was conducted by the Cyprus International Institute for the Environment and Public Health in association with Harvard School of Public Health. A two-stage cluster sample design was used to select a representative sample of students from middle and high schools registered with the Republic of Cyprus in 2005–2006. The study questionnaire consisted of 99 questions and participation in the survey was voluntary. Statistical analyses were performed taking into consideration the specific design of the study and the sample weights associated with each completed questionnaire.

**Results:**

The prevalence of current smoking, defined as having smoked cigarettes on one or more days of the past 30 days, is 13% among boys and 7% among girls in middle schools, and 36% among boys and 23% among girls in high schools. Furthermore, 16% of middle school students and more than 24% of high school students that had never smoked indicated that they are likely to initiate smoking within the next year. Exposure to environmental tobacco smoke is also very high with 91% of students reporting being exposed to smoke in places outside home. In addition, more than 95% of current smokers reported that they had bought cigarettes in a store during the past month and were not refused cigarettes because of their age.

**Conclusion:**

Smoking prevalence among Cyprus middle and high school students is high and there are indications of an increase in the prevalence of smoking among girls over the last few years. Susceptibility rates, exposure to second-hand smoke, and access to and availability of cigarettes to youth are also high and concerning. The present survey indicates that the problem of cigarette smoking among youth in Cyprus is significant and requires collective action immediately.

## Background

Tobacco smoking is a major public health problem worldwide and it is the single most preventable cause of morbidity and mortality in humans [1-3]. Approximately 5 million people die prematurely every year due to tobacco-related diseases, and this rate is projected to double by the year 2020 [4-8].

Adult smokers almost always initiate tobacco use before the age of 18 [9] when they are easily influenced by their peers, social norms, and tobacco advertising. Due to the highly addictive nature of tobacco often simple experimentation of young kids leads to regular smoking well before the age of 18 with more than a third of ever smokers (even one or two puffs) becoming daily smokers before graduating high school and nearly two-thirds becoming regular, daily smokers before they reach the age of 20 [10]. Most young people who smoke regularly during teenage years continue to smoke throughout adulthood with data in the USA suggesting that 80 percent of all adult smokers begin smoking regularly before the age of 18 whereas 90 percent begin smoking regularly before the age of 20 [11]. Initiating smoking at an early age also increases the risk of heavier smoking at a later age [12]. Therefore, tobacco research and prevention programs must focus on the early years of life and include education to prevent youth from initiating smoking. At the same time, smoking in youth (usually defined as at least one cigarette in the last month) is not equivalent to smoking in adults (usually defined as smoking at least one cigarette a day) and the body of evidence of how youth smoking can predict adult smoking is limited. Hence, further research is needed to address the issue of predicting adult smoking based on youth smoking.

The World Health Organization Framework Convention on Tobacco Control (WHO FCTC) reinforces the need for data on adolescent tobacco use by calling on countries to establish surveillance programs of "the magnitude, patterns, determinants, and consequences of tobacco consumption and exposure to tobacco smoke" [13]. Cyprus ratified the WHO FCTC in 2000, thus formally obligating the government of Cyprus to follow the Articles of the WHO FCTC.

In Cyprus limited data exist regarding smoking among youth. A survey conducted by the Ministry of Health in 1997 revealed that 6.5% of all youth 15–19 years old were daily smokers [14] whereas a study in 2003 showed that this number was 14.7% (22.6% for boys and 5.7% for girls) [15]. The European School Survey Project on Alcohol and Other Drugs (ESPAD) reported that 22% (30% for boys and 14% for girls) of 15–16 year-old students surveyed in Cyprus in 2003 had smoked at least once in the past 30 days [16]. In response to the need for additional data, the Cyprus International Institute for the Environment and Public Health in association with Harvard School of Public Health (CII) conducted the Global Youth Tobacco Survey (GYTS) in 2006. The GYTS was developed as a joint project by the WHO, the United States Center for Disease Control and Prevention (CDC), and the Canadian Public Health Association (CPHA). The GYTS collects data on youth smoking behavior, knowledge and attitudes, as well as factors affecting the youth initiation of smoking. It is designed to provide current and comparable data regarding tobacco use among youth around the world, and has already been conducted in more than 151 countries worldwide [5,17-19].

The present study uses data from the 2006 Cyprus GYTS to assess cigarette smoking prevalence (the dominant form of tobacco use in Cyprus) among middle school students (with ages ranging from 12 to 15) and among high school students (with ages ranging from 15 to 18) and study the psychosocial, environmental, and behavioral factors associated with smoking among youth in these ages. The goal of the study is to provide scientific information and evidence in order to help create effective smoking prevention and cessation programs and reduce the smoking rate among youth in Cyprus. Results are reported on the prevalence and factors that influence youth smoking; exposure to second-hand smoke (SHS); access to and availability of tobacco products; exposure to media and advertising; cessation programs; and smoking-related education in Cyprus.

## Methods

The GYTS was conducted in 2006 by the CII, in collaboration with the Harvard School of Public Health, the WHO, the CDC, and the Ministries of Health and Education and Culture of the Republic of Cyprus.

### Population

All middle schools and high schools registered in the academic year 2005–2006 with the Ministry of Education and Culture in Cyprus with a school size of 40 or more students were included in the sampling frame for the GYTS. A two-stage cluster sample design was implemented by the CDC with the goal of selecting a representative sample of students from the Republic of Cyprus drawn from the country's five government-controlled regions, namely Lefkosia, Lemesos, Paphos, Larnaka, and Ammochostos. In the first stage, all middle schools with 1^st^, 2^nd^, and 3^rd ^grades and high schools with 4^th^, 5^th^, and 6^th ^grades were sampled with the probability of selection being equal to the school's enrollment size; in the second stage, classes within chosen schools were selected using a systematic equal-probability sampling with a random start. All students in the selected classes were eligible to participate in the survey.

### Data collection

The study questionnaire was developed based on the Greek translation of the GYTS core questionnaire used in Greece in 2005 [20], and was modified to include questions sensitive to the Cypriot culture, mainly regarding the family role and potential effect of grandparents on tobacco use by their grandchildren. The final study questionnaire consisted of 99 structured response item questions that included demographic information, individual smoking habits, exposure to tobacco advertisements, attitudes and beliefs towards tobacco, exposure to passive smoking, access to tobacco products, school anti-smoking and prevention programs, and habits and attitudes of family and friends regarding tobacco use.

Standardized methodology was followed for the preparation of the questionnaires, the selection of the sample, the field work, and the processing of the data, details of which are described elsewhere [5,17-19]. Permission to conduct the study was obtained from the Ministry of Education and Culture of the Republic of Cyprus prior to the initiation of the field work and data collection. The students' parents were informed about the aims and the methodology of the study in a letter sent to them prior to conducting the survey. The students were briefed about the survey before participating, and they participated on a voluntary basis. The questionnaires and corresponding answer sheets were completed anonymously, and no personal identifier data were collected. The self-administered questionnaires were distributed to the students by trained field workers and were completed in a class group setting during school hours between February 2006 and May 2006.

### Statistical Analyses

Analyses were performed using the Statistical Analysis Software (SAS) version 9.1. The analyses took into account the specific sampling frame and the specific weight associated with each one of the questionnaires. This weight is the product of the inverse of the probability of selecting the school, the inverse of the probability of selecting a classroom within the school, the school-level non-response adjustment factor calculated by school size category (small, medium, large), the class adjustment factor calculated by school, and the student-level non-response adjustment factor calculated by class. Weights are used to reflect the likelihood of sampling of each student and to reduce bias by compensating for differing patterns of non-response. Current smoking is defined as having smoked cigarettes on one or more days of the last 30 days. Qualitative variables are described as percentages together with their corresponding 95% confidence intervals and quantitative variables are described as mean (SD). All statistical tests reported are two-sided, and a p-value < 0.05 is considered to be statistically significant.

## Results

A total of 13,246 students (7,294 from middle schools and 5,952 from high schools) from 90 schools (51 middle schools and 39 high schools) participated in the survey. Out of these, there were 3,651 middle school boys, 3,579 middle school girls, 2,619 high school boys, and 3,312 high school girls that provided gender information (Table 1). The mean age was 13.4 (0.01) for middle school students and 16.2 (0.01) for high school students. The overall school response rate was 96.2% for middle schools and 90.7% for high schools. The student response rate was 92.2% for middle schools and 89.7% for high schools, resulting in an overall response rate (equal to the product of the school and student response rates) of 88.7% for middle schools and 81.4% for high schools.

**Table 1:** Prevalence of tobacco use in youth in Cyprus middle schools and high schools by gender – 2006 Cyprus Global Youth Tobacco Survey

Characteristic	Prevalence (95% Confidence Interval)					
	
	Middle School			High School		
		
	Overall	Boys	Girls	Overall	Boys	Girls
	(n = 7,230)	(n = 3,651)	(n = 3,579)	(n = 5,931)	(n = 2,619)	(n = 3,312)
Age in years – mean (SD)	13.4 (0.01)	13.4 (0.02)	13.3 (0.02)	16.2 (0.01)	16.2 (0.02)	16.2 (0.02)
Ever smoked cigarettes	28.1 (26.2, 29.9)	35.3 (32.9, 37.6)	20.8 (18.3, 23.2)	58.0 (55.8, 60.1)	66.4 (64.6, 68.6)	51.2 (47.7, 54.7)
Ever smokers initiated smoking before age ten	20.0 (18.6, 21.4)	23.4 (20.7, 26.0)	14.3 (11.4, 17.2)	7.8 (6.6, 8.9)	9.9 (7.8, 12.0)	5.6 (4.3, 6.8)
Current tobacco users*	10.5 (9.0, 12.1)	13.6 (11.5, 15.8)	7.3 (5.6, 9.1)	29.2 (27.1, 31.3)	36.8 (34.5, 39.1)	23.1 (20.2, 26.1)
Current cigarette smokers*	9.9 (8.4, 11.4)	12.7 (10.5, 14.9)	7.0 (5.3, 8.7)	28.7 (26.6, 30.8)	35.7 (33.3, 38.0)	23.2 (20.2, 26.2)
Never smokers likely to initiate smoking next year	16.0 (14.7, 17.2)	16.6 (15.2, 18.0)	15.4 (13.6, 17.3)	24.4 (22.8, 26.0)	19.9 (17.6, 22.1)	26.9 (24.6, 29.3)

*Current smoking is defined as having smoked on 1 or more days of the past 30 days

### Prevalence

#### Middle schools (ages 12–15 years)

Table 1 indicates that 28.1% of middle school students had ever smoked cigarettes (even one or two puffs) with boys significantly more likely to do so than girls (35.3% vs. 20.8%, respectively, p < .0001) and that 20.0% of the students initiated smoking before the age of 10 (23.4% for boys and 14.3% for girls, p = 0.0005). Furthermore, 10.5% of middle school students (13.6% of boys and 7.3% of girls, p < .0001) are currently users of some tobacco product, with 9.9% of students being current cigarette smokers; the rate for boys (12.7%) is significantly different than that for girls (7.0%) with p < .0001. Among students that never tried smoking, 16.0% reported that they are susceptible to initiate smoking within the next year, with no statistical difference between boys (16.6%) and girls (15.4%); these susceptibility rates are higher than the current smoking rates.

#### High schools (ages 15–18 years)

The proportion of high school students reporting that they had ever smoked cigarettes is 58.0%, with boys (66.4%) significantly more likely to have done so than girls (51.2%) (Table 1). In addition, 7.8% of the high school students initiated smoking before the age of 10 (9.9% for boys and 5.6% for girls, p = 0.0003). The data also suggest an alarming prevalence of 29.2% currently using some form of tobacco (36.8% for boys and 23.2% for girls, p < .0001); 28.7% of students indicated that they are currently smoking cigarettes, with boys significantly more likely than girls (35.7% vs. 23.2%, respectively). Among students that had never smoked, 24.4% reported that they are susceptible to initiate smoking within the next year, with girls significantly more likely to do so than boys (26.9% vs. 19.9%, respectively, p < 0.0001).

**Figure 1:**
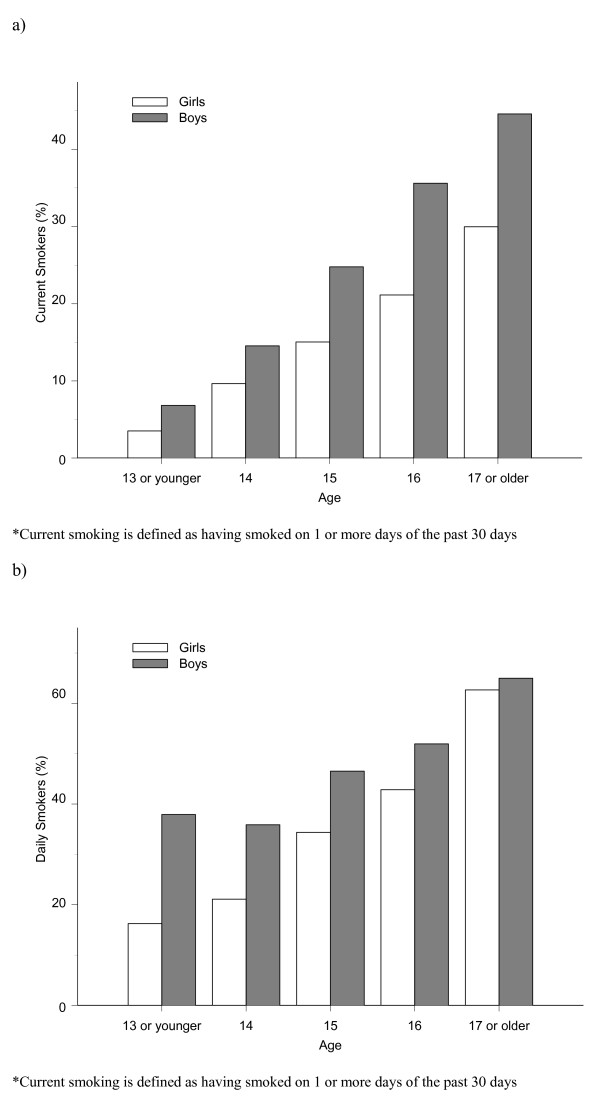
a: Proportion of current cigarette smokers* by age and gender – 2006 Global Youth Tobacco Survey. b: Proportion of students that smoked at least one cigarette every day in the last month among current cigarette smokers* by age and gender – 2006 Global Youth Tobacco Survey

Figure 1a presents the proportion of current smokers by age, for boys and girls, and shows an increasing trend over ages 13 to 17 for both boys and girls. Among students aged 17 or older, the prevalence of smoking is 36.5% (44.6% for boys and 30.0% for girls). Figure 1b, shows the proportion of current smokers by age and gender that smoked daily in the last month and shows a similar increasing trend over the different age groups as that in Figure 1a. Table 2 gives a breakdown of the frequency of smoking (in number of days smoking at least one cigarette in the last month) among current smokers, by gender, separately for middle school and high school students. In high school, almost half of the students that reported to be current smokers also reported smoking daily during the month before the study (52.5% for boys and 43.4% for girls).

**Table 2:** Frequency of smoking (number of days smoking in the last month) among current smokers* by school and gender

Number of days	Proportion (%)					
	
	Middle Schools			High Schools		
		
	Overall (n = 664)	Boys (n = 435)	Girls (n = 229)	Overall (n = 1,580)	Boys (n = 884)	Girls (n = 696)
1–2 days	34.7	31.1	41.3	15.8	13.5	18.6
3–5 days	11.2	8.9	15.5	10.7	9.5	12.1
6–9 days	9.2	8.8	9.8	7.2	6.4	8.3
10–19 days	9.7	9.7	9.7	8.3	8.3	8.3
20–29 days	5.7	5.7	5.6	9.6	9.8	9.3
Everyday	29.5	35.8	18.1	48.4	52.5	43.4

*Current smoking is defined as having smoked on 1 or more days of the past 30 days

### Exposure to SHS

Cypriot students are exposed to SHS at high frequencies (Table 3). Over half of the students (53.5%) have at least one parent that smokes, 84.6% live in homes where others smoke in their presence, and 91.2% are present around others who smoke in places outside the home. Notably, 70.4% of the students "definitely think" that smoke from others is harmful to them and 81.7% of them think that smoking should be banned from public spaces. These rates are significantly higher among non-current smokers than among current smokers with 75.4% of non-current smokers vs. 47.8% of current smokers thinking that second-hand smoke is harmful to them (p < .0001) and 89.8% of non-current smokers vs. only 44.8% of current smokers thinking that smoking should be banned from public places (p < .0001).

**Table 3:** Attributes related to cigarette smoking by current smoking status – 2006 Cyprus Global Youth Tobacco Survey

Attribute	Proportion (95% Confidence Interval)		
	
	Overall (n = 12,955)	Current Smokers* (n = 2,262)	Non-current Smokers (n = 10,693)
**Environmental Tobacco Smoke**			

Have one or more parents who smoke	53.5 (52.6, 54.5)	67.5 (65.0, 70.0)	50.4 (49.3, 51.4)
Exposed to smoke in their home	84.6 (83.8, 85.5)	93.9 (92.9, 94.9)	82.5 (81.6, 83.4)
Around others who smoke in places outside home	91.2 (90.6, 91.8)	95.6 (94.7, 96.6)	90.2 (89.6, 90.9)

**Attitude towards second-hand smoking**			

Think smoke from others is harmful to them	70.4 (69.0, 71.7)	47.8 (44.4, 51.2)	75.4 (74.5, 76.3)
Think smoking should be banned from public places	81.7 (80.6, 82.8)	44.8 (42.3, 47.3)	89.8 (89.1, 90.6)

**Media and Advertising**			

Own an object with a cigarette brand logo	16.5 (15.3, 17.6)	35.0 (32.7, 37.3)	12.5 (11.4, 13.6)
Offered free cigarettes by a tobacco company representative	16.1 (15.1, 17.2)	26.0 (23.6, 28.5)	14.0 (13.1, 15.0)
Saw anti-smoking ads in newspapers/magazines	48.2 (46.7, 49.7)	42.6 (40.3, 45.0)	49.4 (47.8, 51.0)
Saw pro-cigarette ads in newspapers/magazines	62.4 (61.1, 63.7)	63.3 (60.9, 65.6)	62.2 (60.8, 63.6)

**School**			

Had been taught about the dangers of smoking	46.3 (44.5, 48.2)	36.9 (33.9, 39.8)	48.4 (46.4, 50.3)
Had discussed reasons why people their age smoke	42.3 (40.2, 44.4)	37.8 (33.8, 41.8)	43.2 (41.1, 45.3)
Had been taught the effects of tobacco use	46.9 (45.0, 48.7)	40.3 (37.7, 42.9)	48.3 (46.2, 50.4)

*Current smoking is defined as having smoked on 1 or more days of the past 30 days

### Media and Advertising

More than 16% of the students reported owning an item with a cigarette brand logo with significantly more current smokers than non-current smokers owning such an item (35.0% vs. 12.5%, respectively, p < .0001); about an equal proportion (16.1%) reported that they had ever been offered free cigarettes by a tobacco company representative (Table 3). Almost half of the students (48.2%) reported having seen anti-smoking media messages on television and in newspapers and magazines, while an even higher proportion (62.4%) reported having seen pro-cigarette advertisements.

### School

About 40% of students reported that during the past year they had been taught in class about the dangers of smoking and that they had discussed with their teachers and their classmates reasons why people their age smoke (Table 3). About an equal proportion stated that they had been taught in school about the harmful effects of tobacco use. No significant gender differences were observed, but more middle school students than high school students reported being taught in class about the dangers of smoking (54.6% vs. 37.1%, respectively, p < .0001) and discussed the reasons that children their age smoke (45.0% vs. 39.2%, p = 0.006).

### Access and Availability

Among current smokers 61.0% buy their cigarettes in a store (Table 4). Although a law exists in Cyprus prohibiting the sale of cigarettes to children under the age of 18, 95.3% of current smokers reported that they had bought cigarettes in a store in the past month and were not refused purchase because of their age. Table 4 presents these percentages by age categories as well.

**Table 4:** Attributes related to cigarette smoking among current smokers* by age – 2006 Cyprus Global Youth Tobacco Survey

Attribute	Proportion (95% Confidence Interval)					
	
	Overall (n = 2,252)	13 years old or younger (n = 209)	14 years old (n = 263)	15 years old (n = 441)	16 years old (n = 530)	17 years old or older (n = 809)
**Access and Availability**						

Buy cigarettes in a store	61.0 (57.8, 64.2)	44.5 (38.3, 50.8)	46.2 (40.9, 51.5)	57.5 (50.2, 64.7)	61.9 (57.1, 66.7)	69.1 (65.9, 72.3)
Not refused purchase because of their age	95.3 (94.0, 96.6)	85.8 (76.2, 95.5)	89.3 (83.5, 95.1)	94.6 (92.0, 97.2)	95.1 (92.2, 98.1)	97.7 (96.0, 99.3)

**Cessation**						

Want to stop smoking	46.7 (42.7, 50.7)	43.1 (34.6, 51.6)	42.5 (33.6, 51.4)	51.0 (45.0, 57.1)	48.8 (43.7, 53.8)	45.4 (39.7, 51.0)
Tried to stop smoking during the past year	60.9 (57.0, 64.7)	63.1 (53.2, 72.9)	60.9 (54.5, 67.3)	62.7 (56.6, 68.9)	63.5 (53.5, 73.5)	58.2 (55.6, 60.9)
Have ever received help to stop smoking	70.7 (68.1, 73.4)	66.7 (55.3, 78.1)	70.1 (65.6, 74.6)	70.5 (66.1, 74.9)	72.4 (67.3, 77.6)	70.8 (67.0, 74.5)
Have of feel like having a cigarette first thing in the morning	28.2 (26.0, 30.3)	21.5 (14.0, 29.0)	24.8 (16.9, 32.8)	23.8 (17.8, 29.7)	23.9 (18.4, 29.5)	34.2 (30.7, 37.7)

*Current smoking is defined as having smoked on 1 or more days of the past 30 days

### Cessation

Nearly half of current smokers (46.7%) want to stop smoking; 60.9% reported that they tried unsuccessfully to stop smoking during the past year and 70.7% reported that they received help to stop smoking at some point (Table 4). These seem to be quite similar across the different age groups as can be seen in Table 4. At the same time, 28.2% of current smokers admitted to always having or feeling like having a cigarette first thing in the morning, with older students reporting this need at a higher percentage. No statistically significant differences were observed between middle and high schools, nor between genders.

## Discussion

A number of findings from the 2006 Cyprus GYTS raise concerns for the future of tobacco control in Cyprus. First, the prevalence of cigarette smoking among high school boys, defined as smoking on at least one day in the last month, is 35.7%. Furthermore, 30.1% of all 17–18 year old boys and 20.4% of all 15-year or older boys smoke daily. Similar rates were observed in 2003 with the proportion of adult males (15 years old or older) that smoked daily in the last month reported to be 38.1% and the corresponding rate for boys 15–19 years old reported to be 22.6% [15].

Second, and even more concerning, are the smoking rates among high school girls; the prevalence of smoking is 23.2%, with 19.0% of all girls 17–18 years old and 11.8% of all 15-year old or older girls reporting being daily smokers. In 2003, among all adult females 15 years old or older 10.5% were daily smokers, with only 5.7% of girls ages 15–19 being daily smokers [15]. This may suggest a rise in the prevalence of smoking among adult females over the next few years. This is likely to be due to aggressive marketing efforts by tobacco companies that include advertising in fashion magazines targeting younger women as well as more general societal changes with women gaining more independence and smoking becoming more acceptable for them. The increase in the prevalence of smoking among girls and the narrowing of the gap in the corresponding rates between boys and girls is something that has been observed and reported frequently elsewhere [15,21,22].

Third, the susceptibility rates reported are high. Girls in both middle and high school that have never smoked and boys in middle school that have never smoked indicated that they are likely to initiate smoking within the next year at rates higher than the existing prevalence of current smokers. If that holds true then an increase in prevalence rates can be expected, especially among teenage girls in high school whose susceptibility rates are significantly higher than those of high school boys.

When compared with other countries that administered the GYTS, Cyprus has higher current cigarette smoking rates for both boys and girls aged 13–15 than the average of the Eastern Mediterranean region countries sampled (though it has lower rates for current use of other tobacco products) [5,18]. On the other hand, Cyprus' rates are lower compared to the mean rates of the European countries sampled [5,18]. For example, among children 13–15 years old, Cyprus current cigarette smoking rates are 12.3% for boys and 8.2% for girls and are higher compared to Egypt (5.9% and 1.4% for boys and girls, respectively), Lebanon (11.8% and 5.6%), and Saudi Arabia (10.2% and 2.6%) but lower than Bulgaria (26.0% and 39.4%), Czech Republic (29.8% and 32.7%), and Russian Federation (25.9% and 23.9%). [23]. In comparison, Greece, using the GYTS in 2005, reported prevalence rates among children 13–15 years old of 11% for boys and 9% for girls [20]. Furthermore, in 2003, based on the ESPAD, 22% of 15 to 16 year-old students surveyed in Cyprus had smoked in the past 30 days, compared with an average of 35% in the remaining 34 participating countries [16].

It is clear that smoking is an important problem in Cyprus. Conservative estimates suggest that between 600 and 700 people die every year in Cyprus because of smoking (about 12%–13% of about 5,000 deaths of individuals 35 years and older) [24]. In light of the present smoking rates and the increasing trend in smoking among young women, approximately 28,000 of the approximately 200,000 children under 20 years old alive today are projected to die prematurely as adults and many more will suffer from illnesses due to smoking. The government, school officials, health professionals and the society as a whole must intervene as rapidly as possible to counteract these predictions.

Several tobacco control measures exist in Cyprus including regulations regarding the manufacture and sale of tobacco products (e.g. cigarette levels of tar not exceeding 12 mg, no tobacco sales to anyone less than 18 years old), advertising bans and restrictions (e.g. banned in cinemas, regulated in print media), mandatory package warning labels (e.g. 'Smoking Kills' warning covering 32% of the area of one of the two largest surfaces of the outside of the packet), and policies on secondhand smoke (e.g. designated smoking areas, prohibition of smoking in public transportation). However, despite laws that prohibit the sale of tobacco to minors and ban smoking in public places, the present research suggests that these laws are not being observed. Exposure to SHS is extremely high both in the homes of students, where 84.5% report being exposed to smoke, and in other public spaces outside the home, where 91.2% report exposure. SHS is a serious danger even at low levels and is classified by the International Agency for Research on Cancer (IARC) as a known cause of cancer [25]. Measures must therefore be taken to eliminate the exposure of non-smokers (especially children) to passive smoking. The laws banning smoking in public spaces should be strictly enforced and should be tightened where necessary. Also, since tourism is a major industry in Cyprus, it should be made clear that numerous studies across a range of countries have shown that laws that ban smoking in public spaces have no detrimental effects on the businesses of restaurants and bars [26,27]. Hence, the argument that banning smoking in public spaces affects tourism and the sales of restaurants, bars and other similar establishments is flawed. Finally, the laws banning sale of tobacco to minors must be enforced strictly; GYTS results suggest that 95.3% of current smokers that bought cigarettes in a store in the past month were not refused cigarettes because of their age.

The present study is limited by the subjectivity of self-administered questionnaires, introducing the possibility of information bias, as well as some non-response (mainly due to being absent on the day of the survey rather than refusal to participate in the study). Nevertheless, because of the high response rate, the anonymous administration of questionnaires, the good test-retest reliability of similar data, and the openness in Cyprus about the subject of smoking, biases introduced are likely to be minimal [28,29]. The effect of non-response in GYTS was assessed previously and the smoking prevalence reported might in fact be underestimated (even when participation rates are higher than 90%) [30]. This could be the case because often the students that are absent from school on the day of the survey include a substantial number that behave differently than the average student and adopt unhealthy behaviors such as smoking and drinking [30].

Another limitation of the study is the fact that many students in technical schools were not surveyed. However, most of the students in Cyprus attend non-technical schools so we believe that the sample is representative of the whole population (if anything, including technical schools would raise the prevalence rates even higher).

Our recommendations include for the Cyprus government to ensure that the current laws regarding the sale of tobacco to minors and smoking in public spaces are enforced and to further strengthen the existing legislation. In addition, youth programs and anti-tobacco advertising campaigns need to be implemented, and increased professional help for cessation should be made available to persons who want to quit. (One of the few encouraging messages from the survey is that nearly half of the current smokers want to stop smoking.) Public awareness of the dangers of smoking should be promoted through public education campaigns and policy efforts need to be coordinated to address the problem. Further research is also needed in order to understand the complexity of the tobacco problem in Cyprus, the reasons for the high prevalence rates and the increase among females, as well as the specific measures that would effectively reduce tobacco use and its hazardous health effects. The Republic of Cyprus and the Ministry of Health have already adopted the 'Strategic Plan for Tobacco Control in Cyprus' prepared by the Tobacco Research Program of the CII [24]. This plan promises to be an important start towards curtailing the problem in the years to come.

## Conclusion

Though further research is needed in Cyprus in relation to tobacco use and more scientific and updated data should be periodically collected, the GYTS indicates that the problem of cigarette smoking among youth in Cyprus is significant and that collective action needs to be taken immediately.

## Competing interests

The authors declare that they have no competing interests.

## Authors' contributions

PD, GNC, CWW, and NRJ conceived the study, participated in the design, and helped in the development of the methodology. OK supervised and helped in the collection and compilation of the data. CAC performed the statistical analysis, developed the first draft of the manuscript, and revised the manuscript before final submission. HRA and CWW critically revised the manuscript. All authors contributed towards the revision of the manuscript to its final version. All authors read and approved the final manuscript.

## Pre-publication history

The pre-publication history for this paper can be accessed here:


